# Long-Term Follow-Up Outcomes in Women with In Situ/Microinvasive Adenocarcinoma of the Uterine Cervix Undergoing Conservative Treatment—Cervical Adenocarcinoma Study Group Italian Society of Colposcopy and Cervico-Vaginal Pathology

**DOI:** 10.3390/cancers16061241

**Published:** 2024-03-21

**Authors:** Luca Giannella, Giovanni Delli Carpini, Jacopo Di Giuseppe, Camilla Grelloni, Giorgio Bogani, Marco Dri, Francesco Sopracordevole, Nicolò Clemente, Giorgio Giorda, Rosa De Vincenzo, Maria Teresa Evangelista, Barbara Gardella, Mattia Dominoni, Ermelinda Monti, Chiara Alessi, Lara Alessandrini, Angela Guerriero, Alessio Pagan, Marta Caretto, Alessandro Ghelardi, Andrea Amadori, Massimo Origoni, Maggiorino Barbero, Francesco Raspagliesi, Tommaso Simoncini, Paolo Vercellini, Arsenio Spinillo, Giovanni Scambia, Andrea Ciavattini

**Affiliations:** 1 Woman’s Health Sciences Department, Gynecologic Section, Polytechnic University of Marche, 60123 Ancona, Italy; luca.giannella@ospedaliriuniti.marche.it (L.G.); giovanni.dellicarpini@ospedaliriuniti.marche.it (G.D.C.); jacopo.digiuseppe@ospedaliriuniti.marche.it (J.D.G.); c.grelloni@pm.univpm.it (C.G.); 2 Gynecological Oncology Unit, Fondazione IRCCS—Istituto Nazionale Tumori, 20133 Milan, Italy; giorgio.bogani@istitutotumori.mi.it (G.B.); dr.marcodri@gmail.com (M.D.); raspagliesi@istitutotumori.mi.it (F.R.); 3 Gynecologic Oncology Unit, IRCCS—Centro di Riferimento Oncologico di Aviano, 33081 Aviano, Italy; fsopracordevole@cro.it (F.S.); nicolo.clemente@cro.it (N.C.); ggiorda@cro.it (G.G.); 4 Dipartimento Scienze della Salute della Donna, del Bambino e di Sanità Pubblica, UOC Ginecologia Oncologica, Fondazione Policlinico Universitario A. Gemelli IRCCS, 00168 Rome, Italy; rosa.devincenzo@unicatt.it (R.D.V.); mariateresa.evangelista@policlinicogemelli.it (M.T.E.); giovanni.scambia@policlinicogemelli.it (G.S.); 5 Dipartimento di Scienze della Vita e Sanita Pubblica, Università Cattolica del Sacro Cuore, 00168 Rome, Italy; 6 Department of Obstetrics and Gynecology, Fondazione IRCCS Policlinico San Matteo, Università degli Studi di Pavia, 27100 Pavia, Italy; barbara.gardella@gmail.com (B.G.); matti.domino@gmail.com (M.D.); spinillo@smatteo.pv.it (A.S.); 7 Gynecology Unit, Fondazione IRCCS Ca’ Granda Ospedale Maggiore Policlinico, 20122 Milan, Italy; ermelinda.monti@policlinico.mi.it (E.M.); paolo.vercellini@unimi.it (P.V.); 8 UOC Ostetricia Ginecologia, Dipartimento per la Salute della Donna e del Bambino, Azienda Ospedaliera—Università di Padova, 35128 Padova, Italy; chiara.alessi@aopd.veneto.it; 9 Pathological Anatomy Unit, Department of Medicine DIMED, University of Padova, 35128 Padova, Italy; lara.alessandrini@aopd.veneto.it (L.A.); angela.guerriero@aopd.veneto.it (A.G.); 10 UOSD di Patologia Cervico-Vaginale, ULSS 2 Marca Trevigiana, 31100 Treviso, Italy; alessio.pagan@aulss2.veneto.it; 11 Division of Obstetrics and Gynecology, Department of Clinical and Experimental Medicine, University of Pisa, 56126 Pisa, Italy; martacaretto@gmail.com (M.C.); tommaso.simoncini@med.unipi.it (T.S.); 12 Azienda Usl Toscana Nord-Ovest, UOC Ostetricia e Ginecologia, Ospedale Apuane, 54100 Massa, Italy; ghelardi.alessandro@gmail.com; 13 Gynecology Unit, Ospedale di Forlì, 47121 Forlì, Italy; dott.amadori@gmail.com; 14 Department of Gynecology & Obstetrics, Vita Salute San Raffaele University School of Medicine, 20132 Milan, Italy; origoni.massimo@hsr.it; 15 Department of Obstetrics and Gynecology, Asti Community Hospital, 14100 Asti, Italy; barberom@tin.it; 16 Academic Center for Research on Adenomyosis and Endometriosis, Gynecology Unit, Department of Clinical Sciences and Community Health, Università degli Studi di Milano, 20122 Milan, Italy

**Keywords:** adenocarcinoma in situ, microinvasive adenocarcinoma, uterine cervix, follow-up, conservative treatment, human papillomavirus testing

## Abstract

**Simple Summary:**

In situ/microinvasive adenocarcinoma of the uterine cervix represents the majority of cervical glandular lesions and can be treated conservatively. In contrast to squamous lesions, approximately 15–20% of glandular disease may be HPV-negative and therefore associated with a worse prognosis. Furthermore, up to 4% of cases may show recurrence after three years of follow-up. Given the abovementioned risk, knowing the predictive factors of disease recurrence becomes crucial for post-treatment management. In the present study, HPV testing in follow-up was the best predictor for recurrences in women with in situ/microinvasive AC undergoing conservative treatment. So, its use in clinical practice is of pivotal importance. However, attention should be paid to non-high-risk HPV women in follow-up with non-usual-type histopathology, given that it seems to represent a sub-population at increased risk of recurrences.

**Abstract:**

Objective: The present study aimed to assess long-term follow-up outcomes in women with in situ/microinvasive adenocarcinoma (AC) of the uterine cervix treated conservatively. Methods: Retrospective multi-institutional study including women with early glandular lesions and 5-year follow-up undergoing fertility-sparing treatment. Independent variables associated with recurrence were evaluated. Logistic regression analysis and Kaplan–Meier survival analysis with Logrank test were performed. Results: Of 269 women diagnosed with in situ/microinvasive AC, 127 participants underwent conservative treatment. During follow-up, recurrences were found in nine women (7.1%). The only factor associated with recurrence during follow-up was positive high-risk Human Papillomavirus (hr-HPV) testing (odds ratio 6.21, confidence interval 1.47–26.08, *p* = 0.012). HPV positivity in follow-up showed a recurrence rate of 21.7% against 3.8% in patients who were HPV-negative (*p* = 0.002, Logrank test). Among women with negative high-risk HPV tests in follow-up, recurrences occurred in 20.0% of non-usual-type histology vs. 2.1% of usual-type cases (*p* = 0.005). Conclusion: HPV testing in follow-up is of pivotal importance in women with early glandular lesions undergoing conservative treatment, given its recurrence predictive value. However, women who are high-risk HPV-negative in follow-up with non-usual-type histopathology may represent a sub-population at increased risk of recurrences. Further studies should confirm these findings.

## 1. Introduction

Cervical cancer (CC) declined significantly in developed countries thanks to the effectiveness of primary and secondary prevention [1,2,3]. There has been a decline in invasive forms of CC and an increase in in situ stages for squamous lesions [4]. On the contrary, the incidence of cervical glandular lesions, both in situ and invasive forms, is increasing and mainly concerns women aged 30–40 who may have a possible desire to become pregnant [4,5].

Interestingly, in situ/microinvasive adenocarcinoma (AC) of the uterine cervix represents more than 80% of all high-grade glandular lesions [3,5]. Conservative treatment is feasible for these women and shows efficacy similar to definitive treatments [6,7]. However, contrary to what happens for squamous cervical lesions, fertility-sparing treatment provides that the margins of the cone are negative, also in pre-invasive lesions [6,7]. Otherwise, repeated conization is recommended [6,8].

It is well known that cervical glandular lesions can be multifocal, so even with negative margins, the possibility of residual disease is not negligible [8]. In contrast to squamous lesions, approximately 15–20% of glandular disease may be HPV-negative and therefore associated with a worse prognosis [9,10]. Furthermore, up to 4% of cases may show recurrence after three years of follow-up [8]. This finding may be linked to the longer time required for clearance of HPV infection in cervical glandular lesions [8]. Given the issues mentioned above, the follow-up of conservatively treated women must be carefully monitored. Since glandular lesions have a lower incidence than squamous counterparts, several follow-up studies on women with cervical glandular lesions included not large samples with a follow-up varying between 3 and 5 years [11,12,13].

One of the predictive factors most associated with recurrence in cervical glandular lesions is HPV positivity in follow-up [8,14]. A sensitivity of 90% of the HPV test in predicting disease relapse is reported in AIS at the first follow-up visit [14]. However, this also means that relapses may occur in a small proportion of women who are HPV-negative in follow-up.

Based on the above, the objective of the present study was to evaluate the predictive factors of recurrence in women with in situ/microinvasive AC of the uterine cervix during a 5-year follow-up, focusing in particular on the role of both positive and negative HPV tests.

## 2. Materials and Methods

### 2.1. Study Design and Setting

This retrospective multi-institutional study included women treated conservatively with a histological diagnosis of adenocarcinoma in situ (AIS) or microinvasive AC (stage 1A) on cone specimens between January 2012 and December 2017, with a total follow-up of 5 years. All patients with previous conizations, ongoing pregnancy, immunological disease, or undergoing hysterectomy were excluded.

The participating departments are research centers managing women included in both opportunistic and organized cervical cancer screening programs.

### 2.2. Variables

The following variables were collected: age, parity, smoking habit, HPV vaccination status, cone length (mm) (including the overall cone length in case of repeated conizations), conization type, cytology result, HPV testing, lymphovascular space involvement (LVSI), stage (AIS, 1A1, and 1A2), histology (usual type vs. other histology), HPV and Pap test in follow-up, rate of repeated conization for positive ectocervical and/or endocervical margin, lesion location, and recurrence rate [categorized as cervical intraepithelial neoplasia (CIN) 2/3, AIS, cancer]. According to the study period, the histopathological diagnosis of stage 1A refers to the 2014 FIGO staging [15]. Furthermore, given the study period, histological classification refers to the WHO 2014 [16]. Cone length was defined as the distance from the distal or external margin to the proximal or internal margin of the cone specimen [17].

### 2.3. Data Sources/Measurements

All data were retrieved from the electronic database used in our clinics and anonymized before analysis. Fertility-sparing treatment for women with stage AIS or 1A1 without LVSI included conization with negative margins; stage 1A1 with LVSI or 1A2 included conization (including loop electrosurgical excision procedure, laser conization, and cold knife conization) with negative margins + pelvic lymph node dissection [18]. Expert operators performed all conizations in a single surgical step, avoiding fragmentation. Furthermore, all cone specimens had to have clear and interpretable resection margins. The initial treatment was followed by further conization in case of positive margins (ectocervical and/or endocervical). After appropriate counseling, reconization was performed about 30 days after the previous conization. HPV testing included HPV DNA tests like Hybrid Capture 2 and the Cobas 4800 HPV test, including high-risk genotypes 16/18/31/33/35/39/45/51/52/56/58/59/68. Follow-up included standardized planning: HPV and Pap test (co-testing) + colposcopy every six months for three years and then co-testing + colposcopy annually for two years [19,20]. Recurrence rate was measured as the rate of disease relapse during follow-up. The presence of recurrence was based on histological diagnosis. During follow-up, biopsies were performed according to colposcopic appearance. HPV and cytology status during follow-up were considered according to the time of diagnosis of recurrence. Predictive factors associated with disease recurrence were assessed.

### 2.4. Sample Size Calculation

Sample size calculation was performed using the estimation of a confidence interval with a required width for a single proportion based on the study’s primary outcome: recurrence rate in situ/microinvasive ACs. The literature reports a range of disease relapses between 2 and 14% [6,7,21,22,23,24]. We expected a mean value of 8%. With a confidence level of 95% and confidence interval width (2-sided) equal to 10 (±5%), the minimum required sample size should include 118 women.

### 2.5. Ethical Considerations

The Ethical Committee took note of the study (Prot. 270/2023, approved on 8 August 2023). Given the retrospective study design and Italian law, patient consent was not mandatory [25]. This study followed the ethical standards in the 1964 Declaration of Helsinki and its later amendments [26].

### 2.6. Statistical Methods

Categorical variables were expressed as numbers and percentages. The Chi-squared test was used to compare categorical variables. Continuous variables were tested for normal distribution using the Kolmogorov–Smirnov test. The variables were expressed as the median and interquartile range according to the distribution of continuous independent variables. As appropriate, continuous variables were assessed using the Mann–Whitney test. Univariate analysis was performed using logistic regression analysis to find independent recurrence-associated variables. Multivariate logistic regression analysis was performed, including as explanatory variables all the variables that showed a *p*-value ≤ 0.10 in the univariate model. Based on the logistic regression analysis results, follow-up outcomes were measured using the Kaplan–Meier survival analysis with the Logrank test.

MedCalc Statistical Software was used to perform statistical analyses [MedCalc^®^ Statistical Software version 20.305 (MedCalc Software Ltd., Ostend, Belgium; https://www.medcalc.org; 2023, accessed on 28 November 2023)]. A value of *p* < 0.05 was considered statistically significant.

## 3. Results

**Figure 1:**
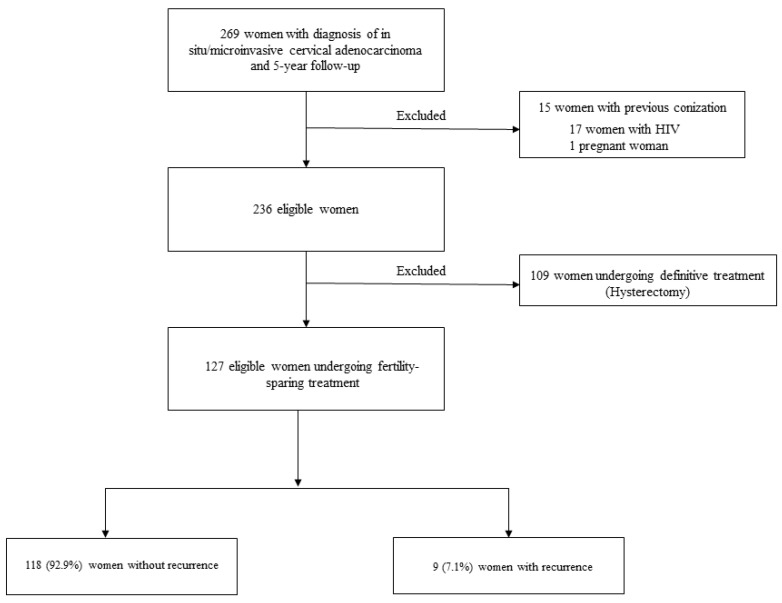
Study flowchart.

The final analysis of this study included 127 women with in situ/microinvasive AC who were undergoing conservative treatment and met the inclusion criteria (Figure 1).

Table 1 reports the characteristics of the patients. The median age was 36 years. The majority of women had never been pregnant (62.2%). The median cone length was 16 mm, while the reconization rate at initial treatment was 28.3%. The high-risk HPV DNA test, available for 92 participants, was positive in about 90% of cases. Women had an AIS cervical lesion in 82% of cases, followed by microinvasive AC 1A1 and 1A2 in 12.6% and 5.5% of cases. Approximately 90% of women had a “usual-type” cervical glandular lesion. Most women had transformation zone type 1 (63.8%), followed by transformation zone type 2 (24.4%) and 3 (11.8%). Positive hr-HPV test in follow-up was found in 23 women (18.1%). There were nine recurrences (7.1%), including AIS lesions, CIN3, and invasive disease (Table 1). Table 1 also shows patient characteristics in women with and without recurrence.

Logistic regression analysis, including all independent variables, showed a significant association between positive hr-HPV test in follow-up and recurrences (odds ratio 6.94, confidence interval 1.7–28.36, *p* = 0.007) (Table 2). The other independent variables showed no significant associations with disease relapse (Table 2). According to the Materials and Methods Section, we performed multivariate analysis, including Pap and HPV test results in follow-up. Positive HPV test in follow-up showed a significant association with recurrences (odds ratio 6.21, confidence interval 1.47–26.08, *p* = 0.012) (Table 2).

**Figure 2:**
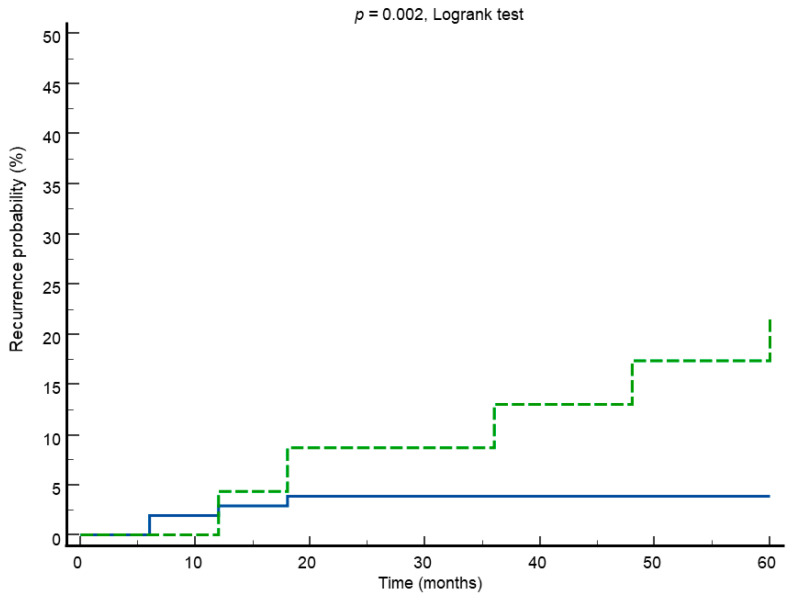
Kaplan–Meier survival curves showing recurrence rate in HPV-positive (green line) vs. HPV-negative (blue line) test in follow-up.

According to the results of univariate analysis, the Kaplan–Meier survival analysis with the Logrank test showed a significant difference in recurrence rate between women with positive vs. negative hr-HPV test in follow-up (Figure 2). HPV positivity in follow-up showed a recurrence rate of 21.7% against 3.8% in patients who were HPV-negative (*p* = 0.002, Logrank test) (Figure 2).

The following data are reported according to the type of transformation zone: in the type 1 transformation zone, the median cone length was 15 mm (CI 10–17 mm); in the type 2 transformation zone, the median cone length was 21 mm (CI 17–31.5 mm); and in the type 3 transformation zone, the median cone length was 26 mm (CI 20.5–34.5 mm).

The median time to recurrence was much earlier in women with a negative HPV test in follow-up than in cases with a positive HPV test [9 vs. 36 months, respectively (*p* = 0.047)]. This apparent paradoxical result can be partly explained by the histopathological distribution of the lesions, as shown below.

According to HPV testing results in follow-up, we looked for a possible sub-population of women most at risk of recurrence by measuring the distribution of the studied variables in HPV-positive and -negative cases. No significant differences were found in women with positive HPV tests in follow-up, including 23 cases. In women with a negative HPV test in follow-up, including 104 cases, a significant difference in the distribution of histopathology was found (Table 3). Among women with negative HPV tests in follow-up, recurrences occurred in 20% of non-usual-type histology vs. 2.1% of usual-type cases (*p* = 0.005) (Table 3).

Based on these results, the distribution of histology type according to pre- and post-treatment HPV test was measured (Table 4). Of 12 non-usual-type histology cases, 10 (83.3%) were found in the hr-HPV negative group during follow-up. The pre-treatment HPV test was negative in 7.4% of usual-type cases vs. 27.3% of non-usual-type histology (including 92 cases). Finally, when we combined pre- and post-treatment HPV with histology (92 cases), we can see that negative/negative cases were present in 18.2% of other histology compared to 7.4% of the usual type (Table 4).

Table 5 shows the detailed events of the nine disease recurrences. It should be noted that in late recurrences, beyond 30 months, all cases reported a previous positive HPV.

## 4. Discussion

The present study showed that HPV positivity in follow-up was the only predictive factor associated with recurrence during a 5-year follow-up. The rate of disease relapse in the HPV-negative group was 3.8%. In the sub-population of women who were HPV-negative durinh follow-up, there was a significantly higher recurrence in cases with other than usual-type histopathology.

Unlike squamous lesions, glandular cervical disease usually has a worse prognosis and a higher recurrence rate [8]. Our results reported a recurrence rate within the range of what is reported in the literature (7.1%) [6,7,21,22,23,24]. As mentioned, in situ/microinvasive AC of the uterine cervix represents the majority of cervical glandular lesions and can be treated conservatively. Indeed, several studies have demonstrated how definitive treatment vs. conization showed no significant differences in recurrence or survival in follow-up studies [7,27]. However, given the abovementioned risk, knowing the predictive factors of disease recurrence becomes crucial for post-treatment management.

It has been reported that a significant predictive factor for recurrence in glandular lesions is represented by follow-up HPV testing [8]. In a recent study, Dostalek et al. retrospectively analyzed the follow-up of 86 cases of microinvasive AC/AIS undergoing conservative treatment during a mean period of 56 months [28]. They confirmed follow-up HPV testing as a strong predictor of recurrence, with no cases of recurrence in the HPV-negative group [28]. In a further study published in 2013, including over 3000 women with CIN1/2/3-AIS cervical lesions, the authors showed how the cumulative risk of recurrence after 1 or 2 negative HPV tests ranged between 2.7% and 3.7% at five years of follow-up [29]. Our data confirmed the positive HPV test in follow-up as the only predictive factor for 5-year recurrence, with a disease relapse rate in HPV-negative cases (3.8%) that did not differ much from that reported by Katki et al. [29].

In an exciting study by Costa et al., the authors reported the diagnostic accuracy of the HPV test in predicting recurrence in 42 patients with AIS treated conservatively [14]. They showed that the sensitivity of the HPV test in the 6- and 12-month follow-up was approximately 90% [14]. We calculated the negative likelihood ratio (LR-) based on their sensitivity and specificity results. This last value is of fundamental importance for predicting the presence of disease when a test is negative [30]. According to their findings [14], with a specificity of 58.3% and a sensitivity of 90%, the LR- is 0.17, representing an excellent but not conclusive value [30]. This is to highlight that a small portion of women with negative HPV tests in follow-up may have a recurrence, as shown by our results.

Given that the HPV-positive population is inherently more at risk of recurrence, in the present study, we wanted to look for any factors stratifying the risk of recurrence in HPV-negative women in order not to miss disease relapses. Interestingly, our results showed that women who were HPV-negative during follow-up with other than usual-type histopathology were at increased risk of recurrence. Recently, the histopathological data in invasive cervical glandular lesions have become increasingly relevant, so much so that in 2018 and 2020, the IECC and the WHO updated the classification of these lesions [31,32,33]. They divided glandular lesions into HPV-associated and HPV-independent lesions. The latter has a worse prognosis, and conservative treatment is not recommended [34]. Unfortunately, given the study period, our data were based on the 2014 WHO classification, which did not consider the above subdivision [10]. Although we did not include cases of HPV-independent adenocarcinoma, we must underline that the 2014 WHO classification in the mucinous AC class includes HPV-associated and HPV-independent types [33]. For example, intestinal ACs can be HPV-associated or HPV-independent, but in the latter case, they are usually found in older women [33]. Furthermore, this concern would only affect a small portion of our sample at stage 1A, including 23/127 cases. So, despite our data showing that cases other than usual-type histology were more present in women who were HPV-negative both in pre-treatment and in follow-up, it is unlikely that the association between negative HPV, non-usual-type histology, and recurrence found in the present study may be due to the inclusion of HPV-independent cases.

A more likely explanation could be the following. We know that cases with negative HPV tests may have HPV-related histopathology. In these cases, HPV guides the initial transformation processes, and it is subsequently lost in the more advanced stages of transformation, where somatic mutations play a predominant role [35,36]. Furthermore, based on the new classifications, it should be underlined that mucinous-type HPV-associated AC (HPVAs) have a worse prognosis than other histotypes in the same class [33]. These data could also explain the earlier recurrence found in our study in HPV-negative cases with histology other than usual-type histology.

From a clinical point of view, our results confirmed the value of HPV testing in follow-up of women with early glandular lesions, given its strong association with recurrences. These data implicitly highlight the importance of HPV vaccination in this population. Although our results did not show a protective role of vaccination, it must be taken into account that vaccinated women included only a tiny portion of our sample (12/127). Therefore, this finding should be considered inconclusive. Another aspect worth mentioning is that women with late disease recurrence (over 30 months) had a previous positive HPV test. This underlines the attention that must be paid to women with a previous positive HPV during the follow-up, even in the event of its subsequent negativity.

Another issue to stress is that out of 23 positive HPV tests in follow-up, 5 later detected the recurrence. Although the positive HPV test has shown its predictive power for recurrences, at the same time, its unsatisfactory specificity is recognized. In this regard, some methods could further increase this value. In an exciting study using CINtec PLUS (CINtec; p16/Ki67 double immunocytochemistry), it was reported that when it was diffusely positive, it was able to achieve accuracy values for glandular lesions of 84.9% (sensitivity), 82.6% (specificity), 83.8% (positive predictive value), and 83.8% (negative predictive value) [37]. An additional test, such as APTIMa mRNA, has shown good accuracy in identifying glandular lesions of the uterine cervix [38]. In this case, detecting HPV RNA E6/E7 expression is a better expression of the virus’s oncogenic activity [39]. Finally, the role of the study of HPV DNA methylation is emerging with satisfactory results in improving the prediction for cervical glandular lesions [40,41]. Based on the above, these methods could have a role in the follow-up of this population.

However, a small portion of disease relapse also occurred in women with high-risk HPV-negative follow-ups. It should be underlined that these cases can be HPV-negative or HPV-positive to non-high-risk genotypes, which have less oncogenic potential [9]. In this sub-population of women at lower risk, further stratification based on histopathology could help avoid missing cases of disease recurrence. Within the AC HPVA class, there may be different prognoses according to histopathology, and this factor becomes even more important in women undergoing conservative treatment. Finally, these data also underline the usefulness of colposcopic evaluation in the follow-up of these patients to perform targeted biopsies on suspicious lesions.

The following limitations of the present study should be considered: (i) its retrospective design; (ii) there was no review of the histology slides; (iii) since our classification is based on WHO 2014, we are not sure that we have excluded all HPV-independent histological cases. However, no histopathologically confirmed gastric, clear cell, endometrioid, or mesonephric type cases were included. On the other hand, it should be emphasized that our sample included a fair number of participants and data from a well-standardized and long-term follow-up.

## 5. Conclusions

HPV testing in follow-up was the best predictor for recurrences in women with in situ/microinvasive AC undergoing conservative treatment. So, its use in clinical practice is of pivotal importance. However, attention should be paid to women who are high-risk HPV-negative in follow-up with non-usual-type histopathology, given that it seems to represent a sub-population at increased risk of recurrences. These data need to be confirmed by further studies.

## Figures and Tables

**Table 1:** Patient characteristics.

Independent Variables	n (%) (Sample Size = 127)	Patients with Recurrence n (%) (Sample Size = 9)	Patients without Recurrence n (%) (Sample Size = 118)
Age (median and interquartile ranges)	36.0 (32.2–39.0)	37 (33.7–40.5)	35.5 (32.0–38.0)
Nulligravid	79 (62.2)	8 (88.9)	71 (60.2)
Smoking habit	28 (22.0)	1 (11.1)	27 (22.9)
Vaccinated	12 (9.4)	2 (22.2)	10 (8.5)
Cone length mm (median and interquartile ranges)	16.0 (12.0–22.7)	15 (14.2–19)	16 (12.0–23.0)
Conization Type			
LEEP	75 (59.1)	6 (66.7)	69 (58.5)
LC	45 (35.4)	3 (33.3)	42 (35.6)
CKC	7 (5.5)	0 (0)	7 (5.9)
Repeated conization	36 (28.3)	3 (33.3)	33 (28.0)
Transformation Zone type			
1	81 (63.8)	6 (66.7)	75 (63.6)
2	31 (24.4)	3 (33.3)	28 (23.7)
3	15 (11.8)	0 (0)	15 (12.7)
Pre-treatment Lesion location			
Ectocervical	32 (25.2)	1 (11.1)	31 (26.3)
Endocervical	38 (29.9)	1 (11.1)	37 (31.4)
Ectocervical + Endocervical	57 (44.9)	7 (77.8)	50 (42.4)
Pre-treatment Pap Test			
Negative	4 (3.1)	0 (0)	4 (3.4)
ASCUS/LSIL	17 (13.4)	2 (22.2)	15 (12.7)
ASCH+	78 (61.4)	5 (55.6)	73 (61.9)
AIS	6 (4.7)	1 (11.1)	5 (4.2)
AGC-NOS	18 (14.2)	1 (11.1)	17 (14.4)
AGC-FN	4 (3.1)	0 (0)	4 (3.4)
Pre-treatment hr-HPV test (available for 92 participants)		(Available for 6 participants)	(Available for 86 participants)
Negative	9 (9.8)	1 (16.7)	8 (9.3)
Positive	83 (90.2)	5 (83.3)	78 (90.7)
Positive LVS	7 (5.5)	1 (11.1)	6 (5.1)
Stage			
1A1	16 (12.6)	2 (22.2)	14 (11.9)
1A2	7 (5.5)	1 (11.1)	6 (5.1)
AIS	104 (81.9)	6 (66.7)	98 (83.1)
Histopathology			
Usual Type	115 (90.6)	7 (77.8)	108 (91.5)
Intestinal Type	6 (4.7)	1 (11.7)	5 (4.2)
Mucinous-NOS	3 (2.4)	1 (11.1)	2 (1.7)
Villoglandular	3 (2.4)	0 (0)	3 (2.5)
Positive hr-HPV in follow-up	23 (18.1)	5 (55.6)	18 (15.3)
Positive Pap test in follow-up			
ASCUS/LSIL	6 (4.7)	0 (0)	6 (5.1)
ASCH+	8 (6.3)	2 (22.2)	6 (5.1)
Negative	113 (89.0)	7 (77.8)	106 (89.8)
Recurrence			
AIS	6 (4.7)	-	-
CIN3	2 (1.6)	-	-
Invasive disease	1 (0.8)	-	-
No recurrence	118 (92.9)	-	-

LEEP: loop electrosurgical procedure; LC: laser conization; CKC: cold knife conization; ASCUS: atypical squamous cells of undetermined significance; LSIL: low-grade squamous intraepithelial lesion; ASCH: Atypical squamous cells—cannot exclude high grade squamous intraepithelial lesion; AGC-NOS: Atypical glandular cells—not otherwise specified; AGC-FN: atypical glandular cells-favor neoplasia; AIS: adenocarcinoma in situ; hr-HPV: high-risk human papillomavirus; LVS: lymphovascular space; CIN: cervical intraepithelial neoplasia.

**Table 2:** Logistic regression analysis showing associations between independent variables and recurrence.

Univariate Analysis				Multivariate Analysis		
Independent Variables	Odds Ratio	Confidence Interval	*p*-Value	Odds Ratio	Confidence Interval	*p*-Value
Age	1.03	0.91–1.16	0.63	-	-	-
Nulligravid	0.18	0.02–1.55	0.12	-	-	-
Smoking habit	0.42	0.05–3.51	0.42	-	-	-
Vaccinated	3.08	0.56–16.88	0.19	-	-	-
Cone length	0.98	0.90–1.06	0.70	-	-	-
Conization Type						
LC	0.82	0.19–3.46	0.78	-	-	-
CKC	-	-	0.99	-	-	-
Repeated conization	1.28	0.30–5.45	0.73	-	-	-
Transformation zone type						
2	1.33	0.31–5.72	0.69	-	-	-
3	-	-	0.99	-	-	-
Pre-treatment Lesion location						
Ectocervical	0.23	0.02–1.96	0.18	-	-	-
Endocervical	0.19	0.02–1.63	0.13	-	-	-
Pre-treatment Pap Test						
Negative/ASCUS/LSIL	1.53	0.27–8.54	0.62	-	-	-
AIS	2.92	0.28–30.02	0.36	-	-	-
AGC-NOS	0.85	0.09–7.83	0.89	-	-	-
AGC-FN	-	-	0.99	-	-	-
Pre-treatment hr-HPV test Available for 92 participants)						
Negative	1.71	0.19–15.50	0.62	-	-	-
Positive LVS	2.33	0.24–21.81	0.45	-	-	-
Stage						
1A1	2.33	0.42–12.7	0.32	-	-	-
1A2	2.72	0.28–26.39	0.38	-	-	-
Histopathology						
Non Usual Type	3.08	0.56–16.88	0.19	-	-	-
Positive hr-HPV in follow-up	6.94	1.7–28.36	0.007	6.21	1.47–26.08	0.012
Positive Pap test in follow-up						
ASCH+	5.33	0.90–31.41	0.06	3.91	0.58–26.39	0.16

LC: laser conization; CKC: cold knife conization; ASCUS: atypical squamous cells of undetermined significance; LSIL: low-grade squamous intraepithelial lesion; AIS: adenocarcinoma in situ; AGC-NOS: Atypical glandular cells—not otherwise specified; AGC-FN: atypical glandular cells–favor neoplasia; hr-HPV: high-risk human papillomavirus; LVS: lymphovascular space; ASCH: Atypical squamous cells—cannot exclude high grade squamous intraepithelial lesion.

**Table 3:** Recurrence rate in HPV-negative cases in follow-up according to histopathology.

Histology in HPV-Negative Follow-Up	Recurrence n (%)	No Recurrence n (%)	*p*-Value
Usual type	2 (2.1)	92 (97.9)	0.005
Other histology	2 (20.0)	8 (80)	

**Table 4:** Distribution of histology type according to pre- and post-treatment HPV test.

Histology *	Pre-Treatment HPV-Negative n (%)	Pre-Treatment HPV-Positive n (%)
Usual type	6 (7.4)	75 (92.6)
Other histology	3 (27.3)	8 (72.7)
Histology	HPV-negative in follow-up n (%)	HPV-positive in follow-up n (%)
Usual type	94 (81.7%)	21 (18.3)
Other histology	10 (83.3)	2 (16.7)
Pre- and post-treatment HPV *	Usual type n (%)	Other histology n (%)
Negative/Negative	6 (7.4)	2 (18.2)
Negative/Positive	0 (0)	1 (9.1)
Positive/Positive	19 (23.5)	1 (9.1)
Positive/Negative	56 (69.1)	7 (63.6)

* Including 92 cases.

**Table 5:** Detailed follow-up events in women with recurrences.

Patients and Histology	Follow-Up Time (Months)							
	6	12	18	24	30	36	48	60
1 Other histology	HPV test negative Colposcopy: negative Biopsy: not performed Histology: -	HPV test negative Colposcopy: positive Biopsy: performed Histology: negative	HPV test negative Colposcopy: positive Biopsy: performed Histology: AIS	-	-	-	-	-
2 Other histology	HPV test negative Colposcopy: negative Biopsy: not performed Histology: -	HPV test negative Colposcopy: positive Biopsy: performed Histology: AIS	-	-	-	-	-	-
3 Usual type	HPV test negative Colposcopy: positive Biopsy: performed Histology: AIS	-	-	-	-	-	-	-
4 Usual type	HPV test negative Colposcopy: negative Biopsy: not performed Histology: -	HPV test negative Colposcopy: negative Biopsy: not performed Histology: -	HPV test negative Colposcopy: negative Biopsy: not performed Histology: -	HPV test negative Colposcopy: positive Biopsy: performed Histology: negative	HPV test positive Colposcopy: negative Biopsy: not performed Histology: -	HPV test negative Colposcopy: negative Biopsy: not performed Histology: -	HPV test positive Colposcopy: positive Biopsy: performed Histology: AIS	-
5 Usual type	HPV test negative Colposcopy: positive Biopsy: performed Histology: AIS	-	-	-	-	-	-	-
6 Usual type	HPV test negative Colposcopy: negative Biopsy: not performed Histology: -	HPV test negative Colposcopy: negative Biopsy: not performed Histology: -	HPV test positive Colposcopy: positive Biopsy: performed Histology: negative	HPV test negative Colposcopy: negative Biopsy: not performed Histology: -	HPV test negative Colposcopy: negative Biopsy: not performed Histology: -	HPV test positive Colposcopy: negative Biopsy: not performed Histology: -	HPV test negative Colposcopy: positive Biopsy: performed Histology: negative	HPV test positive Colposcopy: positive Biopsy: performed Histology: invasive lesion
7 Usual type	HPV test negative Colposcopy: negative Biopsy: not performed Histology: -	HPV test negative Colposcopy: negative Biopsy: not performed Histology: -	HPV test negative Colposcopy: negative Biopsy: not performed Histology: -	HPV test positive Colposcopy: negative Biopsy: not performed Histology: -	HPV test negative Colposcopy: negative Biopsy: not performed Histology: -	HPV test positive Colposcopy: positive Biopsy: performed Histology: CIN3	-	-
8 Usual type	HPV test negative Colposcopy: negative Biopsy: not performed Histology: -	HPV test positive Colposcopy: positive Biopsy: performed Histology: CIN3	-	-	-	-	-	-
9 Usual type	HPV test negative Colposcopy: negative Biopsy: not performed Histology: -	HPV test negative Colposcopy: positive Biopsy: performed Histology: negative	HPV test positive Colposcopy: positive Biopsy: performed Histology: AIS	-	-	-	-	-

HPV: human papillomavirus; AIS: adenocarcinoma in situ; CIN: cervical intraepithelial neoplasia.

## Data Availability

Data are available upon reasonable request.
